# Chemical Composition, Antioxidant and Anti-Enzymatic Activity of Golden Root (*Rhodiola rosea* L.) Commercial Samples

**DOI:** 10.3390/antiox11050919

**Published:** 2022-05-07

**Authors:** Milena Polumackanycz, Pawel Konieczynski, Ilkay Erdogan Orhan, Nurten Abaci, Agnieszka Viapiana

**Affiliations:** 1 Department of Analytical Chemistry, Medical University of Gdansk, Gen. J. Hallera 107, 80-416 Gdansk, Poland; milena.polumackanycz@gumed.edu.pl (M.P.); pawel.konieczynski@gumed.edu.pl (P.K.); 2 Department of Pharmacognosy, Faculty of Pharmacy, Gazi University, Ankara 06330, Turkey; iorhan@gazi.edu.tr (I.E.O.); nurtenabaci@gazi.edu.tr (N.A.); 3 Principal Member of Turkish Academy of Sciences (TÜBA), Vedat Dalokay Street, No. 112, Ankara 06670, Turkey

**Keywords:** *Rhodiola rosea*, antioxidant activity, phenolic composition, elements, neurobiological activity

## Abstract

The aim of the study was to compare the chemical composition of the water and hydromethanolic extracts of *R. rosea* commercial samples in relation to their biological activity. For this purpose, the HPLC method was used for the determination of eleven phenolic compounds and AAS/AES was used for determination of five essential elements. Moreover, the contents of total phenolic, total flavonoid, total phenolic acids, and L(+)-ascorbic acid were determined. The antioxidant activity was assessed by DPPH (2,2-diphenyl-1-picrylhydrazyl) and ABTS radical scavenging activity, ferric-reducing/antioxidant power (FRAP), and cupric-reducing antioxidant capacity (CUPRAC) assays, while the inhibitory activity against AChE and BChE enzymes was determined using Ellman’s method. The results showed that the hydromethanolic extracts of *R. rosea* were richer in phenolic compounds and showed higher antioxidant and neurobiological activity than the water extracts. However, the water extracts gave higher contents of determined elements. Among the individual phenolic compounds gallic acid (2.33 mg/g DW) and sinapic acid (386.44 µg/g DW) had the highest concentrations in the hydromethanolic and water extracts, respectively. Moreover, the most extracts were observed to be more efficient on BChE. Moreover, the correlation analysis indicated a high positive relationship between chemical composition and biological activity in both extracts of *R. rosea*.

## 1. Introduction

The genus *Rhodiola* L. (Crassulaceae) consists of nearly 200 species, where approximately 20 are used in traditional medicine in Asia [1]. One of them is *Rhodiola rosea* L., known as golden root or arctic root. It is a popular herbal medicine with adaptogenic properties and considered safe for human consumption, having no reported adverse effects so far [2,3]. For centuries, its rhizomes have been an important raw material in folk medicine of Scandinavia, Russia, Mongolia, and China as a health-enhancing supplement for stimulating the nervous system, enhancing physical and mental performance, alleviating fatigue, psychological stress, depression, impotence, and preventing high altitude sickness [4,5,6]. Besides the rhizomes and roots show anti-stress, cardioprotective, hepatoprotective, antioxidative, immunomodulatory, anticancer properties, stimulation of the central nervous system as well as increasing cognitive functions such as attention, memory, and learning [5,6,7,8]. The main locations of commercial roots and rhizomes of *R. rosea* are Mountain Altai and in south region of foothill Altai, mainly in the Ust-Kanski, Ust-Koksinski, and Charishki regions [9]. They can be used in the form of crude drug (dried and powdered underground organs) along with its water and alcoholic extracts.

In total, approximately 140 different compounds have been isolated from *R. rosea*, including salidroside, *p*-tyrosol, phenols, flavonoids (e.g., rhodiolin, rhodiosin, tricin, rhodalgin, acetyirhodalgin), and monoterpenes, e.g., rosiridol and rosiridin. The presence of these components may be responsible for the pharmacological effects of *R. rosea* [7]. Moreover, *R. rosea* contains 0.05% essential oils with the main chemical compounds such as *n*-decanol, geraniol, rosiridol, rosiridin, 1,4-menthadien-7-ol, geranyl acetate, benzyl alcohol, and phenylethyl alcohol, which are responsible for the specific rose-like flavor of this raw material [10,11].

Many significant commercial preparations such as food additives, commercial pharmaceutical preparations, dietary supplements, and drinks sold worldwide contain *R. rosea* extracts [2,11]. The content of active ingredients in herbal preparations depends on many factors, such as geographic and climate zone it was grown, in which season, and other conditions it was harvested, how it was dried, extracted, and prepared into final dosage form. According to different studies, some *R. rosea* preparations were effective, while some others not [12,13]. Possible explanation for this might be proposed, when two important circumstances are taken into account such as dose-effect dependence pattern and variety in composition as well as chemistry of active constituents found in different preparations.

An intake of a rich antioxidant diet is associated with a lower risk of chronic diseases. Thus, attention has been paid on the antioxidant capacity of natural products, especially those frequently consumed by people. Different in vitro chemical-based assays have been developed to determine the antioxidant capacity of natural products, e.g., DPPH• scavenging method, trolox equivalence antioxidant capacity (TEAC) assay, or ferric ion reducing antioxidant power (FRAP) assay. Chemical-based methods are useful for screening, as they are low cost, high-throughput, and yield an index value (expressed as equivalents of trolox) that allows comparing and ordering different products. However, considering the diversity of mechanisms an antioxidant compound or mixture can exert in vivo, it is not possible to find a single analytical method to evaluate its antioxidant capacity [14]. It is necessary to apply more than one in vitro chemical-based assay that evaluates different aspects of the reactivity of the compound(s) toward reactive oxygen and nitrogen species (ROS/RNS).

Natural products have already proven to be promising sources of useful acetylcholinesterase (AChE) inhibitors. One of the main benefits is that they have a low toxicity compared to pharmaceutical agents. Cholinesterase inhibitors are the most prescribed drug class currently for the treatment of Alzheimer’s disease (AD), which is a progressive neurodegenerative disorder characterized by memory deficit and behavioral abnormalities particularly in elderly population [15]. Cholinergic hypothesis is one of the accepted mechanisms in the pathology of AD as low level of acetylcholine, which is hydrolyzed by AChE, has been defined in the brains of AD patients [16]. Relevantly, BChE accumulated in the reactive astrocytes may cause worsening the disease [17]. On the other hand, oxidative damage in neurons also negatively contributes to neurodegenerative progress in AD [18]. Moreover, antioxidants support AD therapy by decreasing oxidative damage.

To the best of our knowledge, there is scarce information on the chemical composition and biological activity of *R. rosea* commercial samples. Therefore, the aim of this study was to compare the chemical composition (phenolic compounds and essential elements) of the hydromethanolic and water extracts of commercial samples of *R. rosea* in relation to their antioxidant and acetylcholinesterase (AChE) and butyrylcholinesterase (BChE) inhibitory activity, which is associated with Alzheimer’s disease (AD). Individual phenolic compounds (gallic, protocatechuic, vanillic, caffeic, *p*-coumaric, ferulic, cinnamic and sinapic acids, catechin, rutin, and quercetin) were determined by HPLC technique, while five essential elements (e.g., magnesium, iron, calcium, sodium, potassium) were quantified with the use of AAS/AES technique. The antioxidant activity of *R. rosea* commercial samples was evaluated using four methods, e.g., DPPH and ABTS radical scavenging, ferric-reducing/antioxidant power (FRAP), and cupric-reducing antioxidant capacity (CUPRAC) assays. AChE and BChE inhibitory activity was determined for the ethanolic extracts using Ellman’s method. Moreover, the total content of phenolics, flavonoids, phenolic acids, and L(+)-ascorbic acid was determined in the hydromethanolic and water extracts of commercial *R. rosea* samples.

## 2. Materials and Methods

### 2.1. Plant Materials

Fifteen commercial samples of *R. rosea* compiled in Table 1 were purchased from local supermarkets and pharmacies (Auchan, Gdansk, Poland; Nagietek, Gdansk, Poland; Fragaria, Gdansk, Poland; and Dr Max, Gdansk, Poland). Most of them were in a loose form. Only two samples (no. 10 and 15) were in a form of tablets and one of them (no. 11) was in the form of capsule. All samples were pulverized in a water-cooled Knifetec 1095 grinder (Foss Tecator, Höganäs, Sweden) and the homogenized samples were stored in a light-proof desiccator.

### 2.2. Reagents and Standard Solutions

2,2-Azinobis(3-ethylbenzothiazoline-6-sulfonic acid) diammonium salt (ABTS reagent), 2,2-diphenyl-1-picrylhydrazyl (DPPH reagent), 4-chloro-7-nitrobenzofurazan (NBD-Cl), ammonium acetate, neocuproine, and eleven chemical standards including gallic acid (GA), protocatechuic acid (PRA), catechin (CAT), vanillic acid (VA), caffeic acid (CA), *p*-coumaric acid (*p*CA), ferulic acid (FA), sinapic acid (SIN), rutin (RUT), cinnamic acid (CIN), and quercetin (Q) were purchased from Sigma-Aldrich (St. Louis, MO, USA). In all cases, purity standards exceeded 98%. Aluminium chloride (AlCl_3_) was obtained from Across Organics (Morris Plains, NJ, USA), while HPLC-grade acetonitrile (ACN) from Avantor (Central Valley, PA, USA). Other reagents were obtained from POCh (Gliwice, Poland). The redistilled water was prepared by triple distillation of water in a Destmat^®^ Bi-18 system (Heraeus Quarzglas, Hanau, Germany).

Total phenolic, flavonoid, phenolic acid, L(+)-ascorbic acid contents, and antioxidant activities were determined using a Metertech UV/Vis spectrophotometer (Nankang, Taiwan). Absorbance was measured with the use of a 10 mm quartz cuvette at a suitable wavelength.

### 2.3. Sample Preparation for Analysis of Phenolics

For the hydromethanolic extracts, a 0.5 g of a sample was sonicated with 4 mL of methanol-water mixture (80:20, *v*/*v*) for 20 min at 20 °C using an ultrasonic bath (Emag, Salach, Germany). The suspension was centrifuged in an EBA-20S centrifuge (Hettich, Tuttlingen, Germany) for 15 min at 8000 rpm and the supernatant was transferred into a 20 mL volumetric flask. This procedure was repeated twice and the obtained extracts were combined and diluted up to 20 mL with a mixture of methanol-water (80:20, *v*/*v*).

For the water extracts (infusions) preparation, the sample (2.5 g) was added to 50 mL of boiling distilled water and left to stand at room temperature for 15 min, and then filtered through the Whatman filter paper no. 113 (Sigma-Aldrich, St. Louis, MO, USA).

Before HPLC analysis, hydromethanolic and water extracts of *R. rosea* were filtered through a 0.25 μm nylon filter film (Mecherey, Nagel, Germany), and 20 μL of the filtrate was injected into the HPLC system.

### 2.4. Chromatographic Conditions

Chromatographic separation of phenolics was performed using a Merck-Hitachi LaChrome device (Darmstadt, Germany) equipped with a L-7420 UV-Vis detector, L-7200 autosampler, L-7360 thermostat and D-7000 HPLC System Manager, ver. 3.1 (Merck-Hitachi, Darmstadt, Germany). The method developed for quantitation of ten phenolic compounds was validated by linear range, limit of detection (LOD), limit of quantitation (LOQ), precision, and accuracy according to the procedure described previously [19].

Chromatographic separation was carried out at 40 °C on a Hypersil Gold C18 column (250 × 4.6 mm, 5 µm particles) (Thermo Scientific, Runcorn, UK). The flow rate was set at 1.0 mL/min, the injection volume was 20 µL, and the total run time was 55 min. Gradient elution conditions were used with a mobile phase consisting of a mixture A:B, where A is 0.1% acetic acid in acetonitrile and B is 0.1% acetic acid in water. The gradient profile was t = 0 min, 5% A; t = 5 min, 5% A; t = 40 min, 25% A; t = 45 min, 63% A; t = 50 min, 5% A; t = 55 min, 5% A. The detection wavelengths were set at 280 nm (GA, PRA, CAT, VA, and CIN), 320 nm (CA, *p*CA, FA, and SIN), and 370 nm (RUT and Q). The identification of the phenolic compounds was based on a comparison of the retention time of their standards. Additionally, a selected sample was spiked with the standard compounds and analyzed again.

The validation parameters for HPLC procedure are listed in Table A1. Detailed inspection of the data shows that precision of the HPLC procedure was acceptable, the coefficient of variation (CV) values ranging between 0.25 and 3.94%, and between 0.31 and 7.32%, for intra- and inter-day variations, respectively. For the stability test, retention CV was lower than 1.3% for peak area and 0.8% for retention time. Apart from this, peak areas and retention times of the phenolic compounds were found to be sufficiently stable over 48 h.

### 2.5. Total Phenolic (TPC), Flavonoid (TFC) and Phenolic Acid (TPAC) Contents

The TPC was estimated by the Folin–Ciocalteu method [20]. The Folin–Ciocalteu reagent (0.2 mL) was added to 0.5 mL of diluted sample and after 2 min 7% Na_2_CO_3_ (*w*/*v*) (2 mL) was added. After shaking, the solution was kept at rest for 1 h at room temperature. The absorbance was determined by spectrophotometry at 760 nm after the time of incubation. Gallic acid was chosen as reference to plot the standard curve (linearity range: 0.03–0.15 mg/mL, r = 0.997) and the results were expressed as mg of gallic acid equivalents *per* gram dry weight (DW) of sample (mg GAE/g DW).

TFC was estimated according to the European Pharmacopoeia [21]. Briefly, 0.8 mL of *R. rosea* extract was mixed with 0.1 mL of 5% AlCl_3_ (*w*/*v*) solution and with 1.4 mL of acetic acid and methanol (1:19) mixture. The absorbance was measured at 425 nm after 30 min of incubation in a dark. Quercetin was used as reference to plot the standard curve (linearity range: 4.3–25.7 µg/mL, r = 0.986), and the results were expressed as µg of quercetin equivalents per gram DW of sample (µg QE/g DW).

The procedure was described in the Polish Pharmacopoeia VI [22] was used for TPAC determination with Arnov’s reagent. 0.5 mL of diluted *R. rosea* extract was mixed with 0.2 mL of hydrochloric acid (0.5 M), 0.2 mL of Arnov’s reagent and 0.2 mL of sodium hydroxide (1 M). The absorbance was measured at 490 nm and caffeic acid was used as reference to plot the standard curve (linearity range: 9.6–33.6 µg/mL, r = 0.997). The results were expressed as mg of caffeic acid equivalents *per* gram DW of sample (mg CAE/g DW).

### 2.6. L(+) Ascorbic Acid Content (AA)

The slightly modified method by Abdelmageed [23] was used for ascorbic acid quantification. 0.2 mL of each *R. rosea* extract was mixed with 0.2 mL of NaOH (0.1 M), 0.2 mL of 0.1% (*v*/*v*) acetone 4-chloro-7-nitrobenzofurazane (NBD-Cl) solution and 1.4 mL of 50% (*v*/*v*) aqueous acetone solution. After 30 min, the absorbance was carried out at 582 nm. Ascorbic acid was used as reference to plot the standard curve (linearity range: 0.085–0.699 mg/mL, r = 0.983) and the results were expressed as mg of ascorbic acid *per* gram DW of sample (mg AA/g DW).

### 2.7. Determination of Elements

The contents of macro- and micro-elements were estimated using a VarianSpectrAA 250 Plus absorption/emission spectrometer (250 Plus; Varian Australia). Mg and Fe were determined by atomic absorption spectroscopy, whereas K, Na, and Ca were determined by atomic emission spectroscopy. The appropriate analytical wavelengths for elements were: 285.2 nm (Mg), 422.7 nm (Ca), 248.0 nm (Fe), 589.0 nm (Na), and 766.5 nm (K). The analytical procedures used in the study have been validated. The obtained data of linearity, recovery, LOD, and LOQ were satisfactory [24].

### 2.8. Antioxidant Activity

DPPH assay was performed in accordance with a modified method of Tuberoso et al. [25]. 0.5 mL of diluted extract was added to 2.9 mL of DPPH solution (100 µmol/L) and after 10 min spectrophotometric measuring was carried out at 517 nm. A calibration curve was prepared with trolox (linearity range: 0.01–0.08 mg/mL, r = 0.981) and the results were expressed in mg trolox equivalents *per* gram dry weight of sample (mg TE/g DW).

For ABTS assay, the procedure followed the method of Arnao et al. [26] with some modifications. A volume of 0.3 mL of each diluted extract was added to 2.00 mL of ABTS solution and after 6 min spectrophotometric measuring was carried out at 734 nm. A calibration curve was prepared with trolox (linearity range: 0.02–0.12 mg/mL, r = 0.991) and the results were expressed in mg trolox equivalents *per* gram dry weight of sample (mg TE/g DW).

FRAP assay was performed using the method proposed by Benzie and Strain [27]. A volume of 1.2 mL of each diluted extract was added to 2.25 mL of FRAP solution and, after 30 min, spectrophotometric measuring was carried out at 593 nm. A calibration curve was prepared with ferrous sulphate (linearity range: 280–1089 μmol/L, r = 0.998) and the results were expressed in µmol ferrous ion equivalents *per* gram DW of sample (µmol Fe^2+^/g DW).

The CUPRAC assay was analyzed according to the method of Apak et al. [28] with some modifications. A volume of 0.8 mL of diluted extract was added 1 mL of neocuprine ethanolic solution (7.5 mM), 1 mL of copper chloride solution (0.01 M), 1 mL of ammonium acetate buffer solution (pH = 7.00) and after 30 min spectrophotometric measuring was carried out at 495 nm. A calibration curve was prepared with ascorbic acid (linearity range: 0.012–0.102 mg/mL, r = 0.975) and the results were expressed in mg ascorbic acid equivalents *per* gram DW of sample (mg AA/g DW).

### 2.9. Extract Preparation for Cholinesterase Inhibition Assays

Fifteen commercial samples of *R. rosea* in pulverized form were weighed accurately on a digital balance. The samples were then macerated in 96% ethanol (EtOH, 20 mL) for 3 days at room temperature with periodically shaking by hand. After each sample was filtered through pleated filter paper, the EtOH phases were evaporated under reduced pressure by a rotary evaporator (Büchi, Switzerland) to obtain the crude extracts. The crude extracts were kept in the refrigerator at +4 °C until enzyme inhibition assays were performed. The corresponding percent yields (*w*/*w*) of the extracts are shown in the Table 1.

### 2.10. Cholinesterase Inhibition Microtiter Assays

Inhibitory activity of the extracts against AChE and BChE enzymes was determined using a slightly modified version of Ellman’s method [29]. Electric eel (*Electrophorus electricus*) acetylcholinesterase (Type-VI-S, EC 3.1.1.7, Sigma) and equine serum butyrylcholinesterase (EC 3.1.1.8, Sigma) were employed as the enzyme sources, while acetylthiocholine iodide and butyrylthiocholine chloride (Sigma, St. Louis, MO, USA) were used as reaction substrates. 5,5′-Dithio-bis(2-nitrobenzoic)acid (DTNB, Sigma, St. Louis, MO, USA) was used for the measurement of the cholinesterase activity. Firstly, 140 µL of 0.1 mM sodium phosphate buffer (pH 8.0) was added to the 96-well microplate with a multichannel automatic pipette (Eppendorf Research, Germany) and then 20 µL of the samples/EtOH (negative control) was added at dilutions ranging from 25 to 200 µg/mL. Then, 20 µL of 0.2 M AChE/BChE solution was added by multichannel automatic pipette (Gilson Pipetman, France). After that, it was incubated at room temperature for 10 min. The reaction was started by adding 10 μL of 0.2 M acetylthiocholine iodide/butyrylthiocholine chloride as substrates to the 96-well microplate. Thiol esters used as substrates are hydrolyzed by AChE or BChE to release thiocholine. As a result of the reaction of thiocholine with DTNB, 2-nitro-5-thiobenzoate (TNB) is formed as the yellow-colored product. The formation rate and color intensity of the product, which formed as a result of the reaction, were measured using ELISA microplate reader (Molecular Devices, Spectramax i3x microplate reader with Softmax^®^ Pro Software for Windows 10, San Jose, CA, 95134, USA) at wavelength of 412 nm. Galanthamine hydrobromide (Sigma, USA) was used as the reference in both experiments. All experiments were performed in triplicate. Based on a comparison of rates of enzyme reaction between samples and the blank sample (ethanol in phosphate buffer, pH = 8) using the formula (1–S/E)*100, where E is enzyme activity without test sample and S is enzyme activity with test sample, we determined the percentage of inhibition of AChE and BChE. GraphPad Prism 6.01 was used to compute IC_50_ values.

### 2.11. Statistical Analysis

All samples were analyzed in triplicate. The results were analyzed using the one-way analysis of variance (ANOVA) test, followed by Duncan test. The relationship between *R. rosea* extracts based on the chemical composition, antioxidant activity, and AChE/BChE inhibitory activity was analyzed via Pearson correlation analysis. Statistical data analysis was performed using Statistica 10 software (StatSoft Inc., Tulsa, OK, USA) on the basis of parametric tests with the level of significance of *p* < 0.05.

## 3. Results and Discussion

### 3.1. Total Phenolic (TPC), Flavonoid (TFC), Phenolic Acid (TPAC), and L(+) Ascorbic Acid Contents

The results shown in Table 2 revealed that TPC, TFC, and AA content of the hydromethanolic extracts were significantly higher (*p* < 0.05) than those in water extracts of *R. rosea*, while similar amounts of TPAC were found in both extracts. Moreover, the extract prepared from commercial sample of unknown origin (no. 10) is the lowest in TPC, TFC, TPAC, and AA, while sample no. 4 from Russia was the richest, especially in AA in both extracts.

The literature data on the total phenolic compounds in *R. rosea* extracts are scarce. Hence, the data obtained can also be compared with those found for other *Rhodiola* species. Sist et al. [30] analyzed water extracts of *R. rosea* from Italy and found higher values of TPC (247.2 mg/g extract) than in this study. An explanation behind the variability among the phenolic contents in *R. rosea* extracts could be found due to the different extraction procedures and analytical methods used in each work. In Chinese *R. heterodanta* (Hook. f. & Thomson) Boriss., the values of TPC and TFC obtained by Kumar et al. [31] were 79.21 mg GAE/g and 269.3 mg QE/g, respectively. Tayade et al. [32] determined TPC, TFC, and TPAC of the methanol (112.24 mg GAE/g extract; 30.2 mg QE/g extract; and 39.02 mg GAE/g extract, respectively) and water (59.06 mg GAE/g extract; 17.67 mg QE/g extract; and 16.95 mg GAE/g extract, respectively) extracts of Chinese *R. imbicata* Edgew. To best of our knowledge, there is no previous study investigating AA in *Rhodiola* sp. extracts.

### 3.2. Quantification of Phenolic Compounds

To determine the phenolic compounds in the hydromethanolic and water extracts of commercially available *R. rosea* samples, a simple and reliable HPLC procedure was developed. ANOVA test showed statistically significant differences among the *R. rosea* samples based on the contents of phenolic constituents. As indicated by the data summarized in Table 3, the concentrations of phenolic acids and flavonoids of the hydromethanolic extracts of *R. rosea* commercial samples represent the following order: GA > *p*CA > SIN > Q > CIN > FA > RUT > PRA > CA > VA > CAT. The most abundant phenolic compounds present in the hydromethanolic extracts were GA and *p*CA, while the remaining phenolic compounds were found in relatively lower amounts (their mean content was in µg/g). Moreover, VA was found in 11 samples, CA in 9 samples, RUT in 14 samples, CIN in 10 samples, and Q in 12 samples. VA and CAT were determined at the lowest levels, 64.20 and 18.25 µg/g DW, respectively. In case of the water extracts, the phenolic compounds analysed herein were not present in all samples. SIN and *p*CA were revealed as the most abundant phenolic compounds, being detected in thirteen samples. PRA was found also in thirteen samples, but in very low level, 12.81 µg/g DW. GA and CAT were detected in eleven samples, RUT in eight samples, FA in seven samples, VA and CA in five samples, CIN in only two samples, and Q in only one sample of *R. rosea*. The differences in the content of these compounds could be attributed to climatic and harvesting conditions as well as extraction procedure, which may lead to a loss of bioactive compounds in plants.

Data in the literature on the quantities of individual phenolic compounds in *R. rosea* extracts are scarce. Sist et al. [30] determined phenolic acids and catechin in water extracts of Italian *R. rosea* and found a higher level of VA (22.39 µg/g extract), GA (938.64 µg/g extract), FA (22.47 µg/g extract), and CAT (19.37 µg/g extract) than in this study. However, the content of CIN (215.24 µg/g extract) was compatible with the results obtained herein, while the concentration of CA (92.44 µg/g extract) and *p*CA (68.16 µg/g extract) was about three-fold lower than the concentration of these phenolic acids obtained in this study. Adamczak et al. [33] determined GA and CA in hydromethanolic extracts of Polish *R. rosea*. The authors found that *R. rosea* extract contained 0.47 mg/g DW of GA and 0.10 mg/g DW of CA. In the case of GA, the result was two-fold lower than in this study, while the content of CA was compatible with the results obtained herein. This could be explained by the differences in the growing conditions and in solvents used for extraction in different regions [34]. Moreover, the extraction method, solvent type, and drying process used for the plant material also influence the efficiency of phenolic extraction [35].

### 3.3. Quantification of Elements

Mg, Ca, Fe, K, and Na are important metallic elements for human health. Data regarding elements determination of the hydromethanolic and water extracts obtained from commercial samples of *R. rosea* are presented in Table 4. In general, the water extracts gave higher concentrations of Mg, Ca, Fe, and K (545.79 µg/g DW, 985.25 µg/g DW, 0.80 µg/g DW and 2.89 mg/g DW, respectively) than the hydromethanolic extracts. Considering the hydromethanolic extracts of *R. rosea*, the element with the highest concentration was Na (3.5 mg/g DW), followed by K (0.74 mg/g DW), Ca (158.83 µg/g DW), Mg 148.43 µg/g DW), and Fe (0.12 µg/g DW). However, Fe was determined only in 5 samples (no. 5, 6, 8, 11 and 12), while no significant differences in the concentrations of Na and Ca were noticed. The concentration of elements varies with several factors and may be associated with the type of soil fertilization, agricultural practices, grain genetics, and seed [36]. To the best of the author’s knowledge, determination of elements in *Rhodiola* species has not previously been investigated.

### 3.4. Antioxidant Activity

The antioxidant properties were evaluated by determining DPPH, ABTS, FRAP, and CUPRAC assays in hydromethanolic and water extracts of *R. rosea*. The results are compiled in Table 5. Higher ABTS, FRAP, and CUPRAC assays were obtained for the hydromethanolic extracts (102.45 mg TE/g DW, 109.35 mmol Fe^2+^/g DW, and 17.17 mg AA/g DW, respectively) than those of the water extracts (9.83 mg TE/g DW, 13.12 mmol Fe^2+^/g DW, and 7.50 mg AA/g DW, respectively). It could be related to higher levels of the phenolic compounds in the hydromethanolic extracts. Moreover, the compounds with stronger antioxidant activity in *R. rosea* samples seem to be alcohol-soluble. However, DPPH values for the hydromethanolic and water extracts were on the same level, 59.94 and 59.87 mg TE/g DW, respectively. The hydromethanolic extracts from sample 13 and the water extract from sample 14 were characterized by the highest antioxidant activities, while, among the water extracts, sample 1 showed the lowest level of DPPH, ABTS, and FRAP values. The results varied amongst the methods. The differences may be related to the structure and type of antioxidants detected in *R. rosea* extracts. The findings of this work are in a good agreement with a previous report that the DPPH, ABTS, and FRAP methods detected very different antioxidant capacities in the same samples of propolis varieties [37].

No data on the antioxidant activity of *R. rosea* extracts has been previously reported, excluding Sist et al. [30], who determined FRAP data at level of 4767 μmol Fe^2+^/g extract for water extracts of *R. rosea*. Tayade et al. [32] determined DPPH and ABTS radical scavenging activities of Chinese *R. imbicata* at IC_50_ values of 0.013 mg/mL and 0.016 mg/mL for its methanolic extracts, and 0.04 mg/mL and 0.017 mg/mL for its water extracts, respectively.

### 3.5. AChE and BChE Inhibition Results

EtOH extracts of fifteen commercial samples of *R. rosea* samples are tabulated in Table 6. According to our findings, all of the extracts, except sample no. 10 against BChE, displayed cholinesterase inhibition in varying degrees. Concerning AChE inhibition, the most effective inhibition was caused by sample no. 2 (IC_50_: 3.62 ± 1.22 µg/mL), followed 8 (IC_50_: 4.05 ± 0.70 µg/mL), 4 (IC_50_: 5.22 ± 1.16 µg/mL), 6 (IC_50_: 5.57 ± 0.65 µg/mL), 7 (IC_50_: 6.85 ± 0.44 µg/mL), and 3 (IC_50_: 6.96 ± 0.88 µg/mL), while IC_50_ of galanthamine used as the reference drug was 0.67 ± 0.02 µg/mL. The rest of the samples had IC_50_ values between 8.34 ± 0.57 and 152.2 ± 5.94 µg/mL. On the other hand, the extracts were observed to be more efficient on BChE except sample 10. The strongest inhibition towards BChE was observed by samples 6 (IC_50_: 56.95 ± 4.68 µg/mL), 5 (IC_50_: 71.58 ± 1.85 µg/mL), 3 (IC_50_: 85.77 ± 5.8µg/mL), and 8 (IC_50_: 87.7 ± 3.59 µg/mL), whereas the rest of the samples exhibited higher IC_50_ values than that of galanthamine (IC_50_: 92.34 ± 5.05 µg/mL).

There have been only a few reports available on cholinesterase inhibitory effect of *R. rosea*. Considering our present study, our findings on cholinesterase inhibitory activity of *R. rosae* extracts also contribute to the relevant data on this plant, which has been reported to possess a positive effect on nervous and mental diseases, neuroses, and other neurotic disorders [7]. Among plant phenolic acids, ferulic acid has been shown to display cholinesterase inhibitory activity, which could be suggested as scaffold for novel drug candidates [38]. In an earlier study by Hillhouse et al. [39], AChE inhibitory effect of *R. rosea* extract was found to be 42 ± 3.2% at 10 g/L, while gossypetin-7-O-L-rhamnopyranoside and rhodioflavonoside isolated as two flavonoid glycosides were determined to exert 58 ± 15% and 38 ± 4% inhibition against AChE, respectively, at concentration of 5 g/L. Therefore, phenolic compounds present in the plant might be associated with marked cholinesterase inhibitory effect of the extracts of *R. rosea*. On the other hand, quercetin, a flavonol derivative found abundantly in the hydroalcholic extracts, has been reported to inhibit AChE and BChE at varying levels individually and in many plant extracts [40,41,42]. Besides this, we previously presented that quercetin is concentration-dependent inhibitor of both cholinesterases in a competitive manner. MOreover, quercetin was shown by us to form strong hydrogen bonding to the active gorges of AChE and BChE according to molecular docking simulations [43]. The correlation analysis performed in this study also supported link between BChE inhibition and quercetin, rutin, and cinnamic acid. However, in our earlier study mentioned [42], rutin exhibited insignificant inhibition towards cholinesterases. On the other hand, cinnamic acid has been revealed to be a good pharmacophore for designing new cholinesterase inhibitors, whose derivatives and hybrids have been reported to exert promising inhibition [44,45]. Hence, cinnamic acid could be commented to contribute cholinesterase inhibitory activity of *R. rosea* extracts screened herein. As our correlation analysis indicated a connection between AChE inhibition and vanillic and caffeic acids, vanillic acid detected in *Thunbergia erecta* (Benth.) T.Anderson was reported to inhibit AChE more potently (IC_50_: 30.80 ng/mL) than donepezil (the reference drug, IC_50_: 31.25 ng/mL). Consistently, caffeic acid as well as its hybrids caused to decrease AChE enzyme activity in both in vitro and in vivo methods [46,47]. Consequently, these compounds in particular could be said to contribute to AChE inhibitory action of the active extracts. It should be also noted that chlorogenic acid also possesses cholinesterase inhibitory effect [48,49] and might be acting together with other aforementioned compounds herein within the extracts.

### 3.6. Correlation Analysis

The correlation analysis showed 26 statistically significant correlations (*p* < 0.05) for hydromethanolic extracts. The highest correlations were obtained between FRAP and ABTS assays (0.743), and between TFC and Q (0.751). In the water extracts, 14 statistically significant correlations were found and the highest correlation (0.813) was obtained between TPC and TPAC. Moreover, in the hydromethanolic extracts, DPPH radical scavenging activity results were correlated with FRAP (0.592), while in the water extracts, DPPH and ABTS radical scavenging activity was highly positive, at 0.708. The significant relationships between TPAC and DPPH (0.570) and between TPC and CUPRAC (0.587) for both *R. rosea* extracts imply that the TPAC is one of the main factors responsible for the antioxidant potential of *R. rosea* extracts. Moreover, the significant correlations were found between TPAC and ABTS (0.570), TFC and CUPRAC (−0.551), and TPC and FRAP (0.597) in the hydromethanolic extracts, and between TPC and DPPH (0.667) in water extracts of *R. rosea*. The significant relationships between the antioxidant properties and phenolic compounds imply that the TPC and TPAC are one of the main factors responsible for the antioxidant potential of *R. rosea* samples. This implication was confirmed in the literature [50,51].

The correlation coefficients between the levels of individual phenolic compounds in extracts of *R. rosea* showed that their values are statistically significant (*p* < 0.05) for six and four pairs of constituents in the hydromethanolic and water extracts, respectively. The highest correlation coefficients were found in the pairs of *p*CA-GA (0.695), Q-RUT (0.614) and FA-VA (0.608) in the hydromethanolic extracts, and in the pairs of *p*CA-CAT (0.735), CA-CAT (0.713) in the water extracts. Besides, in the water extracts, *p*CA and CAT were strongly correlated to AA (0.726). In the hydromethanolic extracts, the correlations between GA and *p*CA with TFC were moderately negative, at −0.586 and −0.584, respectively, while correlations between GA and *p*CA with TPAC were moderately positive, at 0.673 and 0.562, respectively. Moreover, RUT was correlated with TPC (0.639) and TFC (0.620), while CA was correlated with AA (0.594) in the hydromethanolic extracts. A strong (0.681) correlation between TPC and TPAC was observed in all analyzed *R. rosea* extracts.

The correlation coefficients between elements and phenolics compounds showed the highest correlation between Fe and AA (0.772), and between Fe and CA (0.707) in the hydromethanolic extracts, and between Fe and TFC (0.809), and K and VA (0.714) in the water extracts. Moreover, high positive relationships were found in the hydromethanolic extracts also for the following pairs: Mg-TPC (0.525), K-TPC (0.602), K-TFC (0.522) and K-RUT (r = 0.605), while in the water extracts for the pairs: Mg-CA (0.572), Ca-TPAC (0.517), Ca-CA (0.622), Na-TFC (0.649) and K-FA (0.667). The correlations between Mg-SIN and Ca-SIN were moderately negative (−0.539) in the hydromethanolic and water extracts, respectively. Besides this, in the water extracts, moderately positive correlations were found between Mg-CUPRAC and Ca-CUPRAC (0.643 and 0.654, respectively).

Moreover, BChE presented a significant (*p* < 0.05) positive correlation with TPAC, TPC, and DPPH (0.787, 0.702, and 0.534, respectively) for water extracts, while AChE was significantly correlated with TFC (−0.805), VA (−0.911), and CA (−0.701). In hydromethanolic extracts, only BChE showed significant (*p* < 0.05) correlation with TPAC (0.573), FRAP (0.604), RUT (0.572), CIN (0.632), and Q (0.639).

## 4. Conclusions

*R. rosea* L. is a popular herbal medicine with adaptogenic properties. This study on the phenolic profile, neurobiological and antioxidant properties of 15 commercially available samples of *R. rosea* revealed that the hydromethanolic extracts were richer in phenolic composition and were characterized by higher values of TPC, TFC, and AA contents than the water extracts. However, the water extracts were richer in studied macro- and micro-elements than the hydromethanolic extracts. Gallic and *p*-coumaric acids were found in higher amounts in the hydromethanolic extracts, and sinapic and *p*-coumaric acids in the water extracts. Moreover, the hydromethanolic extracts have higher antioxidant activity than the water extracts of *R. rosea*. The relationships between analyzed chemical composition and the biological activity of their extracts suggest the crucial role of phenolic constituents as antioxidant agents in *R. rosea* extracts. Overall, the study demonstrated the rich phenolic composition and high biological potential of *R. rosea* accessible to consumers in the form of tea infusion or by incorporating hydromethanolic extracts in antioxidant herbal formulations.

## Figures and Tables

**Table 1:** Characteristics of *Rhodiola rosea* samples used in this study.

No.	Sample Name	Plant Part	Origin	Extract Yields (*w*/*w*)
1.	Rhizome of golden root Ecological tea	Rhizome	Poland	12.88
2.	Rhizome of golden root Ecological tea	Rhizome	Poland	16.11
3.	Rhizome of golden root Ecological tea	Rhizome	Poland	15.06
4.	Rhizome of golden root Herbal tea	Rhizome	Russia	16.92
5.	Superfood golden root powder	Root	China	12.93
6.	Root of golden root	Root	Unknown	22.84
7.	Rhizome of golden root Herbal tea	Rhizome	Russia	19.51
8.	Golden root	Root	Russia	21.14
9.	Golden root (*Rhodiola rosea*) dried herb	Root	Poland	25.81
10.	Golden root	Root	Unknown	15.57
11.	Golden root powder	Root	Russia	13.17
12.	Golden root ECO	Root	Poland	17.24
13.	Golden root	Root	Unknown	14.41
14.	Rhizome of golden root Ecological tea	Rhizome	Poland	16.57
15.	Rhodiola + B tablets	Root	Unknown	10.58

**Table 2:** The quantitative results of total phenolics (TPC), flavonoids (TFC), phenolic acids (TPAC), and L(+)-ascorbic acid (AA) in *R. rosea* commercial samples.

	TPC	TFC	TPAC	AA
	Hydromethanolic extracts			
1	36.27 ± 5.50 ^b^	324.01 ± 6.74 ^d^	1.24 ± 0.06 ^a^	50.87 ± 6.76 ^ab^
2	46.09 ± 4.80 ^bc^	141.90 ± 10.16 ^b^	3.03 ± 0.26 ^c^	105.28 ± 1.26 ^ef^
3	60.17 ± 5.44 ^efg^	141.22 ± 5.48 ^b^	4.11 ± 0.33 ^e^	171.28 ± 0.13 ^h^
4	69.74 ± 1.94 ^gh^	138.42 ± 9.75 ^b^	4.82 ± 0.17 ^f^	213.52 ± 2.35 ^i^
5	64.26 ± 6.33 ^fg^	532.06 ± 6.24 ^i^	3.60 ± 0.46 ^cd^	112.72 ± 1.18 ^fg^
6	61.28 ± 1.94 ^efg^	443.27 ± 4.24 ^g^	3.17 ± 0.98 ^cd^	61.78 ± 1.06 ^abc^
7	56.62 ± 4.01 ^def^	147.94 ± 9.27 ^b^	4.09 ± 0.19 ^e^	132.67 ± 3.89 ^g^
8	75.50 ± 6.25 ^hi^	347.71 ± 1.04 ^e^	3.69 ± 0.35 ^de^	305.22 ± 3.80 ^j^
9	84.74 ± 9.26 ^i^	535.72 ± 3.47 ^i^	3.26 ± 0.24 ^cd^	93.33 ± 2.16 ^def^
10	39.77 ± 2.98 ^b^	116.38 ± 7.32 ^a^	2.15 ± 0.15 ^b^	40.6 ± 4.95 ^a^
11	65.82 ± 6.00 ^fgh^	465.30 ± 8.65 ^h^	2.97 ± 0.09 ^c^	196.91 ± 1.55 ^i^
12	53.13 ± 1.28 ^cde^	301.92 ± 6.19 ^d^	3.30 ± 0.38 ^cd^	82.85 ± 2.33 ^cde^
13	56.66 ± 3.75 ^def^	404.76 ± 9.35 ^f^	4.18 ± 0.03 ^e^	73.61 ± 2.60 ^bcd^
14	46.27 ± 3.03 ^bcd^	274.37 ± 3.58 ^c^	4.03 ± 0.51 ^e^	71.06 ± 1.73 ^bcd^
15	14.64 ± 1.79 ^a^	426.41 ± 3.66 ^g^	1.33 ± 0.15 ^a^	74.46 ± 3.25 ^bcd^
	Water extracts			
1	14.32 ± 0.73 ^a^	210.03 ± 2.70 ^g^	2.01 ± 0.16 ^b^	6.62 ± 1.02 ^ab^
2	20.68 ± 3.86 ^bc^	100.20 ± 3.55 ^b^	2.72 ± 0.53 ^cd^	11.73 ± 2.23 ^d^
3	25.95 ± 1.96 ^de^	110.11 ± 1.58 ^c^	3.55 ± 0.14 ^fg^	12.89 ± 1.32 ^d^
4	28.05 ± 0.67 ^ef^	105.99 ± 1.72 ^bc^	3.92 ± 0.26 ^gh^	15.70 ± 0.60 ^e^
5	22.91 ± 1.49 ^bcd^	285.64 ± 2.52 ^i^	4.03 ± 0.33 ^h^	6.17 ± 0.66 ^a^
6	25.00 ± 5.71 ^cde^	327.55 ± 4.62 ^j^	3.68 ± 0.81 ^fgh^	7.90 ± 0.96 ^bc^
7	26.91 ± 2.27 ^def^	109.15 ± 2.45 ^c^	3.49 ± 0.31 ^f^	8.77 ± 0.14 ^c^
8	23.52 ± 1.44 ^bcde^	280.86 ± 2.28 ^i^	3.33 ± 0.02 ^ef^	8.84 ± 0.36 ^c^
9	30.19 ± 2.80 ^f^	165.23 ± 3.99 ^f^	3.57 ± 0.58 ^fg^	7.62 ± 0.68 ^abc^
10	11.85 ± 3.14 ^a^	79.69 ± 1.84 ^a^	1.35 ± 0.41 ^a^	6.20 ± 0.28 ^ab^
11	27.14 ± 2.06 ^def^	154.60 ± 2.14 ^e^	3.61 ± 0.47 ^fg^	8.84 ± 1.81 ^c^
12	22.37 ± 3.43 ^bcd^	130.74 ± 1.85 ^d^	2.97 ± 0.22 ^d^	7.71 ± 0.10 ^abc^
13	19.43 ± 1.54 ^b^	245.95 ± 1.54 ^h^	3.03 ± 0.21 ^de^	8.43 ± 0.37 ^c^
14	22.37 ± 0.84 ^bcd^	83.58 ± 1.92 ^a^	2.86 ± 0.09 ^d^	10.72 ± 0.91 ^d^
15	12.84 ± 1.31 ^a^	443.85 ± 2.56 ^k^	2.41 ± 0.26 ^c^	8.53 ± 0.38 ^c^

TPC is expressed as mg GEA/g DW; TFC is expressed as µg QE/g DW; TPAC is expressed as mg CAE/g DW; AA is expressed as mg AA/g DW. The results in the same column followed by the same letters do not significantly differ by Duncan’s test (*p* < 0.05).

**Table 3:** The results of individual phenolic compounds in *R. rosea* commercial samples.

	GA	PRA	CAT	VA	CA	*p*CA	FA	SIN	RUT	CIN	Q
Hydromethanolic extracts											
	[mg/g DW]	[μg/g DW]	[μg/g DW]	[μg/g DW]	[μg/g DW]	[mg/g DW]	[μg/g DW]	[μg/g DW]	[μg/g DW]	[μg/g DW]	[μg/g DW]
1	0.97 ± 0.54 ^a^	24.78 ± 7.68 ^a^	4.12 ± 3.91 ^a^	257.48 ± 3.94 ^j^	67.36 ± 1.58 ^c^	0.74 ± 0.24 ^b^	588.87 ± 4.10 ^l^	131.52 ± 2.02 ^c^	77.96 ± 2.77 ^g^	ND	ND
2	2.51 ± 0.25 ^de^	137.65 ± 2.60 ^g^	29.53 ± 9.69 ^fg^	ND	73.98 ± 1.73 ^d^	1.08 ± 0.06 ^bc^	67.54 ± 2.30 ^c^	125.24 ± 2.93 ^b^	50.57 ± 1.39 ^e^	ND	248.20 ± 1.98 ^a^
3	3.23 ± 0.09 ^fg^	331.15 ± 4.97 ^l^	9.65 ± 2.11 ^bc^	36.21 ± 1.75 ^d^	ND	1.97 ± 0.03 ^e^	146.66 ± 3.23 ^f^	844.61 ± 5.25 ^n^	24.79 ± 0.97 ^b^	ND	248.51 ± 1.44 ^a^
4	3.90 ± 0.76 ^h^	134.96 ± 2.20 ^g^	4.94 ± 3.00 ^a^	ND	111.22 ± 2.14 ^f^	3.96 ± 0.71 ^f^	491.68 ± 3.10 ^j^	390.25 ± 3.43 ^i^	ND	367.76 ± 1.83 ^b^	ND
5	2.29 ± 0.02 ^cd^	114.98 ± 4.93 ^f^	28.76 ± 1.05 ^f^	3.74 ± 0.79 ^b^	60.94 ± 1.73 ^b^	0.09 ± 0.01 ^a^	112.61 ± 2.85 ^e^	142.41 ± 2.24 ^d^	127.51 ± 2.85 ^i^	574.66 ± 2.88 ^h^	726.88 ± 5.70 ^h^
6	1.57 ± 0.33 ^b^	72.28 ± 4.71 ^c^	12.34 ± 2.73 ^de^	126.10 ± 2.47 ^i^	ND	1.09 ± 0.22 ^cd^	558.75 ± 3.33 ^k^	561.70 ± 5.43 ^l^	91.27 ± 1.43 ^h^	188.49 ± 2.04 ^a^	387.40 ± 2.70 ^e^
7	3.29 ± 0.52 ^g^	100.79 ± 9.45 ^d^	48.13 ± 0.94 ^h^	49.33 ± 2.41 ^f^	ND	2.84 ± 0.45 ^f^	158.81 ± 1.52 ^g^	121.84 ± 4.63 ^a^	59.42 ± 0.49 ^f^	522.23 ± 7.76 ^g^	255.73 ± 1.53 ^b^
8	1.68 ± 0.30 ^b^	143.09 ± 1.23 ^h^	11.39 ± 2.44 ^cd^	32.14 ± 1.57 ^c^	347.88 ± 2.17 ^h^	1.08 ± 0.16 ^cd^	96.80 ± 1.39 ^d^	433.28 ± 4.84 ^j^	330.65 ± 2.71 ^l^	426.51 ± 6.81 ^e^	397.67 ± 2.44 ^f^
9	1.61 ± 0.09 ^b^	199.75 ± 8.68 ^k^	3.10 ± 0.55 ^a^	45.25 ± 1.14 ^e^	146.29 ± 3.63 ^g^	1.93 ± 0.19 ^e^	64.63 ± 1.01 ^c^	234.17 ± 3.71 ^f^	424.95 ± 3.49 ^m^	453.96 ± 6.92 ^f^	649.66 ± 3.07 ^g^
10	2.00 ± 0.43 ^bc^	113.76 ± 9.37 ^f^	8.09 ± 1.29 ^b^	ND	ND	1.08 ± 0.28 ^bc^	16.27 ± 0.12 ^a^	317.46 ± 4.26 ^h^	32.42 ± 1.70 ^c^	ND	ND
11	1.91 ± 0.06 ^bc^	106.01 ± 1.47 ^e^	10.92 ± 2.45 ^cd^	58.75 ± 2.57 ^g^	ND	0.37 ± 0.08 ^a^	607.93 ± 3.01 ^m^	391.80 ± 5.51 ^i^	206.17 ± 2.11 ^j^	65.43 ± 1.68 ^c^	282.83 ± 1.89 ^c^
12	3.68 ± 0.48 ^gh^	155.62 ± 7.41 ^i^	30.82 ± 1.28 ^g^	ND	ND	1.46 ± 0.08 ^d^	41.15 ± 1.12 ^b^	279.99 ± 3.32 ^g^	32.17 ± 1.52 ^c^	ND	254.95 ± 2.36 ^b^
13	1.93 ± 0.11 ^bc^	42.60 ± 2.52 ^b^	13.19 ± 1.55 ^e^	83.32 ± 4.66 ^h^	55.37 ± 0.87 ^a^	0.03 ± 0.01 ^a^	115.13 ± 2.95 ^e^	161.89 ± 3.92 ^e^	253.59 ± 2.93 ^k^	186.44 ± 3.05 ^a^	327.16 ± 3.71 ^d^
14	2.81 ± 1.45 ^ef^	162.05 ± 3.55 ^j^	55.34 ± 5.76 ^i^	7.51 ± 3.67 ^a^	104.09 ± 2.93 ^e^	1.91 ± 0.47 ^e^	189.88 ± 4.97 ^h^	535.93 ± 4.14 ^k^	40.01 ± 0.33 ^d^	371.23 ± 1.27 ^b^	256.62 ± 1.88 ^b^
15	1.60 ± 0.28 ^b^	38.85 ± 4.94 ^b^	3.40 ± 2.69 ^a^	6.40 ± 2.85 ^a^	55.85 ± 0.88 ^a^	0.12 ± 0.04 ^a^	236.37 ± 5.60 ^i^	684.19 ± 5.82 ^m^	19.53 ± 1.85 ^a^	193.49 ± 3.56 ^d^	245.87 ± 2.04 ^a^
Water extracts											
	[μg/g DW]	[μg/g DW]	[μg/g DW]	[μg/g DW]	[μg/g DW]	[μg/g DW]	[μg/g DW]	[μg/g DW]	[μg/g DW]	[μg/g DW]	[μg/g DW]
1	ND	6.02 ± 1.02 ^a^	5.70 ± 0.44 ^d^	25.51 ± 0.23 ^d^	514.15 ± 5.21 ^d^	152.97 ± 2.39 ^f^	8.76 ± 0.66 ^e^	414.84 ± 3.07 ^i^	ND	ND	ND
2	359.10 ± 3.39 ^f^	22.18 ± 1.10 ^e^	3.62 ± 0.11 ^c^	ND	496.75 ± 2.83 ^a^	290.87 ± 2.49 ^i^	5.40 ± 0.92 ^a^	300.38 ± 1.58 ^g^	ND	ND	ND
3	ND	11.22 ± 0.12 ^b^	15.70 ± 1.08 ^f^	ND	502.99 ± 3.97 ^c^	413.06 ± 3.77 ^k^	ND	181.55 ± 2.68 ^e^	ND	ND	ND
4	34.53 ± 0.77 ^a^	14.93 ± 0.26 ^c^	11.83 ± 0.16 ^e^	ND	495.81 ± 4.96 ^a^	440.62 ± 4.22 ^l^	ND	174.92 ± 2.54 ^d^	ND	ND	ND
5	475.96 ± 5.03 ^h^	ND	0.36 ± 0.06 ^a^	ND	482.62 ± 2.41 ^b^	ND	ND	77.44 ± 1.22 ^b^	17.89 ± 0.97 ^c^	ND	ND
6	ND	7.07 ± 0.14 ^a^	1.22 ± 0.46 ^ab^	18.23 ± 0.78 ^c^	ND	109.15 ± 2.15 ^b^	3.46 ± 0.01 ^c^	383.13 ± 3.05 ^h^	87.89 ± 1.03 ^g^	ND	ND
7	588.55 ± 2.93 ^j^	24.81 ± 0.64 ^e^	1.60 ± 0.30 ^b^	ND	ND	358.51 ± 3.07 ^j^	ND	144.19 ± 4.65 ^c^	ND	ND	ND
8	ND	16.00 ± 0.03 ^cd^	1.17 ± 0.21 ^ab^	12.14 ± 0.46 ^a^	ND	137.87 ± 1.68 ^d^	5.26 ± 0.52 ^a^	683.35 ± 4.35 ^j^	33.29 ± 1.35 ^f^	211.86 ± 1.32 ^b^	ND
9	ND	15.14 ± 0.69 ^c^	0.40 ± 0.05 ^a^	1.79 ± 0.05 ^b^	ND	123.16 ± 1.97 ^c^	4.82 ± 0.06 ^d^	784.41 ± 5.77 ^l^	28.20 ± 1.61 ^e^	ND	241.75 ± 1.57
10	168.20 ± 2.37 ^d^	5.54 ± 0.23 ^a^	ND	ND	ND	224.79 ± 2.50 ^h^	ND	774.32 ± 7.53 ^l^	96.74 ± 1.12 ^h^	ND	ND
11	53.23 ± 0.79 ^b^	6.81 ± 0.51 ^a^	ND	ND	ND	45.32 ± 1.76 ^a^	12.84 ± 0.44 ^f^	64.76 ± 1.81 ^a^	21.75 ± 1.38 ^d^	ND	ND
12	301.90 ± 2.99 ^e^	10.93 ± 0.44 ^b^	0.24 ± 0.04 ^a^	ND	ND	108.96 ± 1.51 ^b^	ND	291.48 ± 2.44 ^f^	ND	ND	ND
13	402.18 ± 3.88 ^g^	7.08 ± 0.76 ^a^	0.93 ± 0.03 ^ab^	14.33 ± 0.75 ^a^	ND	ND	0.62 ± 0.03 ^b^	ND	12.12 ± 0.84 ^b^	144.09 ± 1.46 ^a^	ND
14	508.53 ± 3.81 ^i^	18.78 ± 0.54 ^d^	ND	ND	ND	179.24 ± 1.96 ^g^	ND	748.99 ± 6.96 ^k^	ND	ND	ND
15	142.76 ± 1.32 ^c^	ND	ND	ND	ND	144.55 ± 1.20 ^e^	ND	ND	1.93 ± 0.21 ^a^	ND	ND

The results in the same column followed by the same letters do not significantly differ by Duncan’s test (*p* < 0.05); ND: not detectable.

**Table 4:** The results of determination of elements in *R. rosea* samples.

	Mg	Ca	Fe	Na	K
	[µg/g DW]	[µg/g DW]	[µg/g DW]	[mg/g DW]	[mg/g DW]
Hydromethanolic extracts					
1	208.58 ± 2.25 ^g^	175.88 ± 5.85 ^b^	ND	3.62 ± 0.35 ^ab^	1.02 ± 0.16 ^fg^
2	144.00 ± 3.72 ^cde^	140.02 ± 4.32 ^b^	ND	1.30 ± 0.40 ^a^	0.52 ± 0.01 ^bc^
3	123.30 ± 1.04 ^c^	147.12 ± 7.69 ^b^	ND	2.18 ± 0.79 ^ab^	0.64 ± 0.08 ^cd^
4	129.76 ± 2.75 ^c^	165.65 ± 9.31 ^b^	ND	1.83 ± 0.08 ^a^	0.61 ± 0.03 ^bcd^
5	160.62 ± 2.35 ^def^	167.80 ± 9.04 ^b^	0.21 ± 0.02 ^ab^	1.12 ± 0.04 ^a^	0.76 ± 0.02 ^de^
6	187.65 ± 4.45 ^fg^	474.76 ± 7.53 ^d^	0.14 ± 0.01 ^a^	2.58 ± 0.29 ^ab^	1.10 ± 0.23 ^g^
7	143.59 ± 5.26 ^cde^	162.07 ± 1.22 ^b^	ND	1.97 ± 0.03 ^ab^	0.60 ± 0.07 ^bcd^
8	172.60 ± 1.16 ^ef^	172.96 ± 2.65 ^b^	1.17 ± 0.22 ^c^	4.07 ± 0.40 ^cd^	0.82 ± 0.09 ^e^
9	208.57 ± 5.89 ^g^	152.08 ± 5.37 ^b^	ND	7.71 ± 4.48 ^e^	1.51 ± 0.11 ^h^
10	188.62 ± 2.62 ^fg^	148.09 ± 2.96 ^b^	ND	6.01 ± 0.00 ^de^	0.49 ± 0.02 ^bc^
11	161.31 ± 4.00 ^def^	145.28 ± 2.57 ^b^	0.49 ± 0.05 ^b^	4.85 ± 0.26 ^cd^	0.85 ± 0.01 ^ef^
12	136.74 ± 4.99 ^cd^	246.46 ± 4.93 ^c^	0.11 ± 0.09 ^a^	4.93 ± 1.46 ^cd^	0.77 ± 0.05 ^de^
13	161.86 ± 1.58 ^def^	28.43 ± 3.99 ^a^	ND	2.53 ± 0.68 ^a^	0.46 ± 0.05 ^ab^
14	67.85 ± 5.57 ^b^	28.87 ± 4.91 ^a^	ND	3.09 ± 0.30 ^bcd^	0.58 ± 0.15 ^bc^
15	31.35 ± 2.12 ^a^	27.01 ± 2.80 ^a^	ND	4.68 ± 1.18 ^cd^	0.33 ± 0.07 ^a^
Water extracts					
1	810.61 ± 4.99 ^h^	951.72 ± 3.74 ^bc^	0.51 ± 0.30 ^bc^	1.70 ± 0.14 ^bc^	4.84 ± 0.24 ^f^
2	549.43 ± 3.83 ^c^	1268.41 ± 5.28 ^cd^	0.52 ± 0.26 ^bc^	2.18 ± 0.09 ^cd^	2.63 ± 0.30 ^bc^
3	630.38 ± 2.43 ^de^	1258.78 ± 6.63 ^cd^	0.45 ± 0.22 ^abc^	1.60 ± 0.13 ^b^	2.39 ± 0.05 ^b^
4	572.57 ± 3.92 ^cd^	1584.43 ± 5.05 ^d^	0.29 ± 0.10 ^ab^	1.32 ± 0.17 ^ab^	2.38 ± 0.03 ^b^
5	768.05 ± 3.66 ^gh^	1643.57 ± 7.96 ^d^	1.00 ± 0.24 ^d^	1.60 ± 0.56 ^b^	2.96 ± 0.05 ^c^
6	711.93 ± 2.83 ^fg^	1434.8 ± 5.83 ^d^	1.47 ± 0.16 ^e^	1.48 ± 0.23 ^ab^	4.03 ± 0.44 ^e^
7	680.32 ± 3.37 ^ef^	1423.22 ± 4.53 ^d^	0.73 ± 0.18 ^cd^	1.81 ± 0.22 ^bc^	2.46 ± 0.16 ^b^
8	335.14 ± 2.35 ^a^	501.25 ± 2.50 ^ab^	0.84 ± 0.06 ^cd^	2.43 ± 0.09 ^d^	2.99 ± 0.12 ^c^
9	424.56 ± 3.26 ^b^	648.02 ± 3.26 ^ab^	0.21 ± 0.00 ^ab^	1.25 ± 0.26 ^ab^	3.9 ± 0.44 ^e^
10	551.87 ± 2.06 ^cd^	475.93 ± 3.72 ^a^	ND	0.97 ± 0.17 ^a^	1.85 ± 0.10 ^a^
11	454.17 ± 4.80 ^b^	662.82 ± 3.87 ^ab^	1.06 ± 0.24 ^d^	1.29 ± 0.57 ^ab^	3.37 ± 0.03 ^d^
12	339.04 ± 2.95 ^a^	598.89 ± 4.07 ^ab^	0.1 ± 0.07 ^a^	1.57 ± 0.03 ^b^	2.51 ± 0.10 ^b^
13	567.65 ± 4.37 ^cd^	762.13 ± 3.60 ^ab^	0.46 ± 0.03 ^abc^	1.39 ± 0.18 ^ab^	2.67 ± 0.16 ^bc^
14	446.67 ± 4.11 ^b^	615.02 ± 5.96 ^ab^	0.23 ± 0.12 ^ab^	1.84 ± 0.61 ^bc^	2.63 ± 0.04 ^bc^
15	344.45 ± 5.27 ^a^	949.74 ± 3.78 ^bc^	2.28 ± 0.46 ^f^	4.67 ± 0.44 ^e^	1.77 ± 0.10 ^a^

The results in the same column followed by the same letters do not significantly differ by Duncan’s test (*p* < 0.05). ND: not detectable.

**Table 5:** The results of DPPH, ABTS, FRAP, and CUPRAC *in R. rosea* commercial samples.

	DPPH	ABTS	FRAP	CUPRAC
	[mg TE/g DW]	[mg TE/g DW]	[µmol Fe^2+^/g DW]	[mg AA/g DW]
Hydromethanolic extracts				
1	17.14 ± 1.15 ^a^	106.57 ± 9.99 ^gf^	65.19 ± 4.56 ^b^	9.49 ± 0.98 ^b^
2	20.61 ± 2.01 ^a^	67.71 ± 2.11 ^ab^	75.77 ± 5.02 ^bc^	14.18 ± 1.01 ^cd^
3	62.31 ± 1.80 ^cd^	81.27 ± 7.25 ^bc^	84.23 ± 5.95 ^c^	33.55 ± 0.45
4	67.50 ± 3.99 ^def^	100.61 ± 3.72 ^def^	120.07 ± 7.29 ^ef^	33.20 ± 0.97 ^f^
5	65.27 ± 1.84 ^cde^	88.68 ± 3.69 ^cd^	109.19 ± 9.01 ^e^	2.35 ± 0.10 ^a^
6	66.73 ± 5.76 ^def^	93.96 ± 7.21 ^cde^	104.71 ± 1.05 ^de^	2.34 ± 0.38 ^a^
7	62.67 ± 2.17 ^cd^	67.39 ± 6.63 ^ab^	90.02 ± 3.37 ^cd^	27.35 ± 0.57 ^e^
8	60.11 ± 1.63 ^c^	88.45 ± 6.63 ^cd^	132.37 ± 8.34 ^f^	25.07 ± 0.84 ^e^
9	78.92 ± 5.53 ^h^	108.78 ± 8.06 ^f^	166.40 ± 6.82 ^g^	16.08 ± 2.39 ^d^
10	59.77 ± 0.86 ^c^	55.62 ± 2.28 ^a^	65.21 ± 4.25 ^b^	10.95 ± 2.39 ^bc^
11	71.83 ± 1.46 ^fg^	91.24 ± 5.93 ^cd^	114.68 ± 1.48 ^e^	9.08 ± 0.95 ^b^
12	64.50 ± 6.75 ^cd^	80.23 ± 4.89 ^bc^	80.24 ± 7.71 ^bc^	16.28 ± 3.91 ^d^
13	71.92 ± 4.31 ^efg^	195.41 ± 5.94 ^g^	218.79 ± 5.35 ^h^	25.13 ± 3.60 ^e^
14	76.99 ± 7.76 ^gh^	198.69 ± 2.03 ^g^	165.88 ± 4.34 ^g^	17.25 ± 1.09 ^d^
15	52.87 ± 1.37 ^b^	112.20 ± 1.92 ^f^	47.43 ± 8.02 ^a^	15.21 ± 3.95 ^d^
Water extracts				
1	37.55 ± 3.16 ^a^	4.88 ± 0.33 ^a^	7.71 ± 1.24 ^abc^	9.24 ± 0.56 ^ef^
2	51.53 ± 3.06 ^bc^	9.02 ± 1.29 ^bcd^	11.87 ± 4.53 ^de^	6.00 ± 0.73 ^bc^
3	59.01 ± 2.86 ^d^	10.60 ± 0.61 ^bcde^	11.37 ± 1.49 ^de^	8.13 ± 0.94 ^de^
4	60.17 ± 5.73 ^def^	11.11 ± 0.29 ^cde^	14.52 ± 4.29 ^ef^	11.94 ± 2.17 ^gh^
5	62.72 ± 1.88 ^defg^	10.40 ± 1.08 ^bcde^	10.26 ± 2.34 ^cd^	14.03 ± 1.58 ^h^
6	63.04 ± 5.17 ^defg^	7.07 ± 0.92 ^ab^	10.25 ± 5.83 ^cd^	10.75 ± 1.33 ^fg^
7	70.73 ± 4.59 ^i^	9.47 ± 1.39 ^bcd^	8.64 ± 1.01 ^abcd^	8.83 ± 2.03 ^ef^
8	63.61 ± 5.09 ^efg^	7.84 ± 0.82 ^ab^	14.33 ± 1.57 ^ef^	5.60 ± 0.38 ^bc^
9	68.92 ± 1.11 ^hi^	11.51 ± 1.23 ^cde^	9.16 ± 1.92 ^bcd^	6.29 ± 1.25 ^cd^
10	52.98 ± 4.28 ^c^	8.30 ± 0.80 ^bc^	22.23 ± 5.23 ^g^	4.54 ± 0.40 ^bc^
11	65.37 ± 2.36 ^gh^	12.04 ± 1.00 ^de^	6.72 ± 1.20 ^ab^	12.11 ± 0.90 ^g^
12	64.00 ± 3.95 ^fg^	11.74 ± 0.94 ^cde^	5.52 ± 0.37 ^a^	4.72 ± 0.17 ^bc^
13	59.76 ± 4.02 ^de^	12.40 ± 0.55 ^de^	29.66 ± 1.56 ^h^	4.79 ± 1.13 ^bc^
14	70.55 ± 0.87 ^i^	13.27 ± 0.37 ^e^	19.89 ± 0.43 ^g^	3.98 ± 1.30 ^b^
15	48.04 ± 1.30 ^b^	7.74 ± 0.49 ^ab^	15.88 ± 2.18 ^f^	1.59 ± 0.47 ^a^

The results in the same column followed by the same letters do not significantly differ by Duncan’s test (*p* < 0.05).

**Table 6:** AChE and BChE inhibition% and IC_50_ values of EtOH extracts of *R. rosea* commercial samples.

	Cholinesterase Inhibition (Inhibition % ± S.D. ^a^ at 200 µg/mL ^b^)	
Sample No.	AChE	BChE
1	93.29 ± 0.35 (IC_50_: 16.05 ± 0.76 µg/mL)	54.18 ± 0.34 (IC_50_: 159.45 ± 0.64 µg/mL)
2	90.71 ± 4.27 (IC_50_: 3.62 ± 1.22 µg/mL)	58.51 ± 2.03 (IC_50_: 133.15 ± 0.07 µg/mL)
3	89.98 ± 1.15 (IC_50_: 6.96 ± 0.88 µg/mL)	71.23 ± 4.62 (IC_50_: 85.77 ± 5.8µg/mL)
4	86.1 ± 0.88 (IC_50_: 5.22 ± 1.16 µg/mL)	72.88 ± 1.66 (IC_50_: 95.46 ± 4.96 µg/mL)
5	78.85 ± 1.87 (IC_50_: 8.34 ± 0.57 µg/mL)	76.68 ± 0.28 (IC_50_: 71.58 ± 1.85 µg/mL)
6	73.39 ± 1.27 (IC_50_: 5.57 ± 0.65 µg/mL)	93.04 ± 4.70 (IC_50_: 56.95 ± 4.68 µg/mL)
7	89.25 ± 2.18 (IC_50_: 6.85 ± 0.44 µg/mL)	80.29 ± 1.45 (IC_50_: 114.45 ± 3.32 µg/mL)
8	84.32 ± 1.96 (IC_50_: 4.05 ± 0.70 µg/mL)	93.88 ± 1.69 (IC_50_: 87.7 ± 3.59 µg/mL)
9	85.52 ± 0.62 (IC_50_: 9.19 ± 1.40 µg/mL)	87.65 ± 2.69 (IC_50_: 111.57 ± 8.53 µg/mL)
10	88.7 ± 3.63 (IC_50_: 24.8 ± 0.84 µg/mL)	34.19 ± 2.50
11	82.38 ± 2.87 (IC_50_: 11.2 ± 1.15 µg/mL)	68.24 ± 0.70 (IC_50_: 85.51 ± 0.49 µg/mL)
12	93.29 ± 1.70 (IC_50_: 26.34 ± 2.50 µg/mL)	63.39 ± 3.04 (IC_50_: 149.9 ± 3.54 µg/mL)
13	91.01 ± 0.57 (IC_50_: 13.41 ± 1.82 µg/mL)	82.52 ± 4.05 (IC_50_: 123.85 ± 4.17 µg/mL)
14	88.36 ± 0.59 (IC_50_: 21.21 ± 3.61 µg/mL)	72.19 ± 3.02 (IC_50_: 116.0 ± 5.66 µg/mL)
15	58.47 ± 0.73 (IC_50_: 152.2 ± 5.94 µg/mL)	60.45 ± 2.06 (IC_50_: 173.3 ± 5.23 µg/mL)
Ref. ^c^	97.57 ± 2.59 (IC_50_: 0.67 ± 0.02 µg/mL)	89.56 ± 0.80 (IC_50_: 92.34 ± 5.05 µg/mL)

^a^ S.D.: Standard deviation (n: 3). ^b^ Final concentration. ^c^ Galanthamine at 200 µg/mL.

## Data Availability

Data is contained within the article.

## Appendix A

**Table A1:** Parameters of calibrations for the phenolics standards used.

Analytes	Regression Equation	Linearity [µg/mL]	R^2^	LODs [µg/mL]	LOQs [µg/mL]	Recovery [%]
**GA**	y = 24,923x + 80,484	43.6–218	0.999	2.45	8.44	97.43
**PRA**	y = 47,573x + 12,323	40–200	0.996	1.34	4.23	95.36
**CAT**	y = 34,601x + 1768	40–200	0.996	3.64	9.32	92.11
**VA**	y = 31,673x + 10,265	42.2–212	0.995	1.65	4.21	102.34
**CA**	y = 25,354x − 57,202	43.2–216	0.997	2.75	7.53	91.54
***p*CA**	y = 11,674x + 17,339	43.2–216	0.995	4.23	8.54	93.66
**FA**	y = 24,405x + 58,515	42.8–214	0.994	1.74	3.78	105.31
**SIN**	y = 23,485x + 32,642	43.6–218	0.993	1.31	3.14	91.43
**RUT**	y = 47,624x + 11,078	43.2–216	0.994	2.64	6.98	95.02
**CIN**	y = 5430x + 39,394	41.2–206	0.982	2.86	7.53	96.35
**Q**	y = 38,124x − 38,307	43.2–216	0.970	5.24	11.31	97.21

y is the peak area. x refers to the concentration of compounds (µg/mL).

## References

[1] Bawa A.S., Khanum F. (2009). Anti-inflammatory activity of *Rhodiola roseaea* second-generation adaptogen. Phytother. Res..

[2] Chiang H.M., Chen H.C., Wu C.S., Wu P.Y., Wen K.C. (2015). *Rhodiola* plants: Chemistry and biological activity. J. Food Drug Anal..

[3] Zhou T., Zheng J., Zhou Y., Li S., Gan R., Li H. (2015). Chemical components and bioactivities of *Rhodiola rosea*. Inst. J. Tradit. Med..

[4] Galambosi B., Galambosi Z., Hethelyi E., Szöke E., Volodin V., Poletaeva I., Llijina I. (2010). Importance and quality of roseroot (*Rhodiola rosea* L.) growing in the European North. Z. Arznei-Gewurzpfla.

[5] Bykov V.A., Zapesochnaya G.G., Kurkin V.A. (1999). Traditional and biotechnological aspects of obtaining medicinal preparations from *Rhodiola rosea* L. (a review). Pharm. Chem. J..

[6] Limanagi F., Biagioni F., Busceti C.L., Polzella M., Fabrizi C., Fornai F. (2020). Potential antidepressant effect of *Scutellaria baicalensis*, *Hericium erinaceus* and *Rhodiola rosea*. Antioxidants.

[7] Panossian A., Wikman G., Sarris J. (2010). Roseroot (*Rhodiola rosea*): Traditional use, chemical composition, pharmacology and clinical efficacy. Phytomedicine.

[8] Edwards D., Heufelder A., Zimmermann A. (2012). Therapeutic effects and safety of *Rhodiola rosea* extract WS^®^ 1375 in subjects with life-stress symptoms–results of an open-label study. Phytother. Res..

[9] Saratikov A.S., Krasnov E.A. (2004). Rhodiola rosea (Golden root).

[10] Brown R.P., Gerbarg P.L., Ramazanov Z. (2002). *Rhodiola rosea*: A phytomedicinal overview. Herb. Gram..

[11] Evstatieva L., Todorova M., Antonova D., Staneva J. (2010). Chemical composition of essential oils of *Rhodiola rosea* L. of three different origins. Pharmacogn. Mag..

[12] Colson S.N., Wyatt F.B., Johnston D.L., Autrey L.D., Fitz Gerald Y.L., Earnest C.P. (2005). *Cordyceps sinensis*- and *Rhodiola rosea*-based supplementation in male cyclists and its effect on muscle tissue oxygen saturation. J. Strength Cond. Res..

[13] Earnest C.P., Morss G.M., Wyatt F., Jordan A.N., Colson S., Church T.S., Fitzgerald Y., Autrey L., Jurca R., Lucia A. (2004). Effects of a commercial herbal-based formula on exercise performance in cyclists. Med. Sci. Sports Exerc..

[14] López-Alarcón C., Denicola A. (2013). Evaluating the antioxidant capacity of natural products: A review on chemical and cellular-based assays. Anal. Chim. Acta.

[15] Lane C.A., Hardy J., Schott J.M. (2018). Alzheimer’s disease. Eur. J. Neurol..

[16] Saxena M., Dubey R. (2019). Target enzyme in Alzheimer’s disease: Acetylcholinesterase inhibitors. Curr. Top. Med. Chem..

[17] Jasiecki J., Targońska M., Wasąg B. (2021). The role of butyrylcholinesterase and iron in the regulation of cholinergic network and cognitive dysfunction in Alzheimer’s disease pathogenesis. Int. J. Mol. Sci..

[18] Simunkova M., Alwasel S.H., Alhazza I.M., Jomova K., Kollar V., Rusko M., Valko M. (2019). Management of oxidative stress and other pathologies in Alzheimer’s disease. Arch. Toxicol..

[19] Viapiana A., Struck-Lewicka W., Konieczynski P., Wesolowski M., Kaliszan R. (2016). An approach based on HPLC-fingerprint and chemometrics to quality consistency evaluation of *Matricaria chamomila* L. commercial samples. Front. Plant Sci..

[20] Singleton V.L., Rossi J.A. (1965). Colorimetry of total phenolics with phosphomolybdic-phosphotungstic acid reagents. Am. J. Eno. Vitic..

[21] (2002). Birkenblätter–Betulae Herba 4.00. European Pharmacopoeia.

[22] Polish Pharmaceutical Society (2002). Polish Pharmacopoeia VI.

[23] Abdelmageed O.H., Khashaba P.Y., Askal H.F., Saleh G.A., Refaat I.H. (1995). Selective spectrophotometric determination of ascorbic acid in drugs and foods. Talanta.

[24] Konieczynski P., Arceusz A., Wesolowski M. (2016). Essential elements and their relations to phenolic compounds in infusions of medicinal plants acquired from different European regions. Biol. Trace Elem. Res..

[25] Tuberoso C.I.G., Rosa A., Bifulco E., Melis M.P., Atzeri A., Pirisi F.M., Dessi M.A. (2010). Chemical composition and antioxidant activities of *Myrtus communis* L. berries extracts. Food Chem..

[26] Arnao M., Cano A., Acosta M. (2001). The hydrophilic and lipophilic contribution to total antioxidant activity. Food Chem..

[27] Benzie I.F.F., Strain J.J. (1996). The ferric reducing ability of plasma (FRAP) as a measure of “antioxidant power”: The FRAP assay. Anal. Biochem..

[28] Apak R., Güçlü K., Demirata B., Ozyürek M., Celik S.E., Bektasoglu B., Berker K.I., Ozyurt D. (2007). Comparative evaluation of varioustotal antioxidant capacity assays applied to phenolic compounds with the CUPRAC assay. Molecules.

[29] Ellman G.L., Courtney K.D., Andres V., Featherstone R.M. (1961). A new and rapid colorimetric determination of acetylcholinesterase activity. Biochem. Pharmacol..

[30] Sist P., Tramer F., Lorenzon P., Urbani R., Vrhovsekc U., Bernareggi A., Sciancalepore M. (2018). *Rhodiola rosea*, a protective antioxidant for intense physical exercise: An in vitro study. J. Funct. Foods.

[31] Kumar R., Tayade A., Chaurasia O.P., Sunil H., Singh S.B. (2010). Evaluation of antioxidant activities and total phenol and flavonoid content of the hydro-alcoholic extracts of *Rhodiola* sp. Pharmacogn. J..

[32] Tayade A.B., Dhar P., Sharma M., Chauhan R.S., Chaurasia O.P., Srivastava R.B. (2013). Antioxidant capacities, phenolic contents, and GC/MS analysis of *Rhodiola imbricata* Edgew. root extracts from Trans-Himalaya. J. Food Sci..

[33] Adamczak A., Buchwald W., Gryszczyńska A. (2016). Biometric features and content of phenolic compounds of roseroot (*Rhodiola rosea* L.). Acta Soc. Bot. Pol..

[34] Ristivojevic P.M., Tahir A., Malfent F., Milojkovic Opsenica D., Rollinger J.M. (2019). High-performancethin-layer chromatography/bioautography and liquid chromatography-mass spectrometry hyphenatedwith chemometrics for the quality assessment of *Morus alba* samples. J. Chromatogr. A.

[35] Dent M., Dragovic-Uzelac V., Penic M., Brncic M., Bosiljkov I., Levaj B. (2013). The effect of extraction solvents, temperature and time on the composition and mass fraction of polyphenols in dalmatian wild sage (*Salvia officinalis* L.). Food Technol. Biotechnol..

[36] Pedro A.R.N., de Oliveira E., Cadore S. (2006). Study of the mineral content of chocolate flavoured beverages. Food Chem..

[37] Andrade J.K.S., Denadai M., de Oliveira C.S., Nunes M.L., Narain N. (2017). Evaluation of bioactive compounds potential and antioxidant activity of brown, green and red propolis from Brazilian northeast region. Food Res. Int..

[38] Singh Y.P., Rai H., Singh G., Singh G.K., Mishra S., Kumar S., Srikrishna S., Modi G. (2021). A review on ferulic acid and analogs based scaffolds for the management of Alzheimer’s disease. Eur. J. Med. Chem..

[39] Hillhouse B.J., Ming D.S., French C.J., Towers G.H.N. (2004). Acetylcholine esterase inhibitors in *Rhodiola rosea*. Pharm. Biol..

[40] Olennikov D.N., Kashchenko N.I., Chirikova N.K., Akobirshoeva A., Zilfikarov I.N., Vennos C. (2017). Isorhamnetin and quercetin derivatives as anti-acetylcholinesterase principles of marigold (*Calendula officinalis*) flowers and preparations. Int. J. Mol. Sci..

[41] Orhan I.E. (2021). Cholinesterase inhibitory potential of quercetin towards Alzheimer’s disease—A promising natural molecule or fashion of the day?—A narrowed review. Curr. Neuropharmacol..

[42] Tenfen A., Vechi G., Cechinel-Zanchett C.C., Lorenzett T.S., Reginato-Couto C.E., Siebert D.A., Vitali L., Micke G., Klein-Júnior L.C., Cechinel Filho V. (2021). Phenolic profile by HPLC-ESI-MS/MS of six Brazilian *Eugenia* species and their potential as cholinesterase inhibitors. Nat. Prod. Res..

[43] Khan M.T., Orhan I., Senol F.S., Kartal M., Sener B., Dvorská M., Smejkal K., Slapetová T. (2009). Cholinesterase inhibitory activities of some flavonoid derivatives and chosen xanthone and their molecular docking studies. Chem. Biol. Interact..

[44] Estrada M., Herrera-Arozamena C., Perez C., Vina D., Romero A., Morales-Garcia J.A., Perez-Castillo A. (2016). New cinnamic-*N*-benzylpiperidine and cinnamic-*N*,*N*-dibenzyl (*N*-methyl) amine hybrids as Alzheimer-directed multitarget drugs with antioxidant, cholinergic, neuroprotective and neurogenic properties. Eur. J. Med. Chem..

[45] Ghafary S., Ghobadian R., Mahdavi M., Nadri H., Moradi A., Akbarzadeh T., Najafi Z., Sharifzadeh M., Edraki N., Moghadam F.H. (2020). Design, synthesis, and evaluation of novel cinnamic acid-tryptamine hybrid for inhibition of acetylcholinesterase and butyrylcholinesterase. DARU.

[46] Chao X., He X., Yang Y., Zhou X., Jin M., Liu S., Cheng Z., Liu P., Wang Y., Yu J. (2012). Design, synthesis and pharmacological evaluation of novel tacrine-caffeic acid hybrids as multi-targeted compounds against Alzheimer’s disease. Bioorg. Med. Chem. Lett..

[47] Anwar J., Spanevello R.M., Thomé G., Stefanello N., Schmatz R., Gutierres J., Vieira J., Baldissarelli J., Carvalho F.B., da Rosa M.M. (2012). Effects of caffeic acid on behavioral parameters and on the activity of acetylcholinesterase in different tissues from adult rats. Pharmacol. Biochem. Behav..

[48] Kwon S.H., Lee H.K., Kim J.A., Hong S.I., Kim H.C., Jo T.H., Park Y.I., Lee C.K., Kim Y.B., Lee S.Y. (2010). Neuroprotective effects of chlorogenic acid on scopolamine-induced amnesia via anti-acetylcholinesterase and anti-oxidative activities in mice. Eur. J. Pharmacol..

[49] Katalinic M., Rusak G., Domacinovic Barovic J., Sinko G., Jelic D., Antolovic R., Kovarik Z. (2010). Structural aspects of flavonoids as inhibitors of human butyrylcholinesterase. Eur. J. Med. Chem..

[50] Gonçalves S., Gomes D., Costa P., Romano A. (2013). The phenolic content and antioxidant activity of infusions from Mediterranean medicinal plants. Ind. Crop. Prod..

[51] Doshi P., Adsule P., Banerjee K., Oulkar D. (2015). Phenolic compounds, antioxidant activity and insulinotropic effect of extracts prepared from grape (*Vitis vinifera* L.) by products. J. Food Sci. Technol..

